# Data related to anaerobic digestion of bioplastics: Images and properties of digested bioplastics and digestate, synthetic food waste recipe and packaging information

**DOI:** 10.1016/j.dib.2019.103990

**Published:** 2019-05-27

**Authors:** Wei Zhang, Francisco Torrella, Charles J. Banks, Sonia Heaven

**Affiliations:** a Faculty of Engineering and Physical Sciences, University of Southampton, Southampton SO17 1BJ, UK; b Departamento de Genética y Microbiologia, Universidad de Murcia Spain

**Keywords:** Bioplastic, Anaerobic digestion, Biodegradation, Plastic film, Food waste, Co-digestion

## Abstract

The data presented in this article are related to the research article entitled ‘Degradation of some EN13432 compliant plastics in simulated mesophilic anaerobic digestion of food waste’ (W. Zhang, S. Heaven, C. Banks, 2018). Zhang et al., 2018. They include quantification of residual materials from preparation of a synthetic food waste feedstock; photographic images of the physical appearance of the test plastics after prolonged exposure to microbial degradation in a continuously-operated anaerobic digestion trial; microscopic images of selected plastics after anaerobic biodegradation; test data and results for a Biochemical Methane Potential assay for the plastics; analytical data for potentially toxic elements in the plastics; and values for residual biogas potential of the digestate. Additional data on experimental methods is given, including a recipe for a synthetic food waste specifically designed for use in anaerobic digestion simulation studies; and details on adjustment of calculations after amendment of the digestate sampling methodology used in the main study.

**Table:** Specifications table

Subject area	*Engineering*
More specific subject area	*Anaerobic digestion of bioplastics*
Type of data	*Tables, images (photographic and microscopic), graphs*
How data was acquired	*Laboratory experimental (in-house anaerobic digestion equipment), laboratory analytical (gas composition by gas chromatography using a Varian CP* 3800 GC*) and microscopy (Olympus BX53 with phase contrast system and digital camera DP72; Leica TCS SP2 confocal laser scanning microscope).*
Data format	*Analyzed*
Experimental factors	*Methylene blue staining for some microscopic samples*
Experimental features	*Batch biochemical methane potential tests and semi-continuous trials in mesophilic continuously-stirred tank reactors as described in**[1]*
Data source location	*Faculty of Engineering and the Environment, University of Southampton, Southampton SO17* 1BJ*, UK*
Data accessibility	*Data is with this article*
Related research article	*Zhang, W., Heaven, S. and Banks, C., 2018. Degradation of some EN13432 compliant plastics in simulated mesophilic anaerobic digestion of food waste. Polymer Degradation and Stability. 147, 76–88,*https://doi.org/10.1016/j.polymdegradstab.2017.11.005*,**[1]*

**Table:** 

**Value of the data** • Visual data on physical appearance of plastics after digestion may be used in comparative evaluation of degradation performance and in assessment of mechanisms • Microscopy images may offer researchers supporting evidence for theories on degradation and attack mechanisms • Biochemical methane potential (BMP) values, Potentially toxic element (PTE) content and residual biogas potential provide comparative data for alternative methods and other research • Synthetic food waste recipe can be used in other investigations • Data on reject materials from the synthetic food waste can be used in research on food-related packaging waste generation rates.

## 1 Data

The data presented in this document are related to a work on degradation of some EN13432 compliant plastics in simulated mesophilic anaerobic digestion of food waste [1].

### 1.1 Residual materials from synthetic food waste recipe

**Fig. 1:**
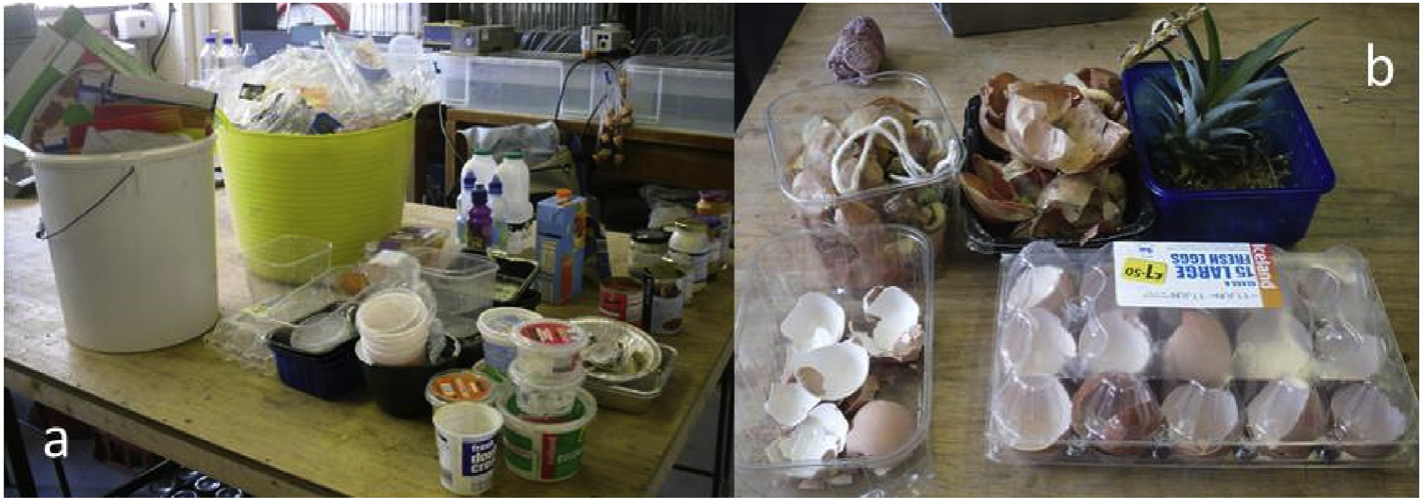
Items rejected during SFW preparation: (a) Packaging materials, (b) Materials not put through macerator.

During preparation of the synthetic food waste (SFW) used in the trial, the packaging material in which it came was separated (Fig. 1) and weighed. The total unsorted weight of material including all food items and packaging was 101.836 kg, of which the rejected packaging stream made up 4.604 kg. Plastic film made up 774 g or 0.76% of the total unsorted weight, while solid plastics (trays, pots and bottles) made up a further 880 g or 0.86%, giving a plastics total of 1.62% on a wet weight basis (Table 1). Further details of the mixed SFW and card packaging (CP) feedstock used in the trial are given in section 2.1.

**Table 1:** Food and packaging streams from SFW materials.

Item	Weight (g)	% of total unsorted weight (including food items)
Plastic bottles	140	0.14%
Plastic trays	446	0.44%
Plastic containers/pots	294	0.29%
Subtotal solid plastic	880	0.86%
Plastic film	774	0.76%
Total plastic (not including Tetra pak components)	1654	1.62%
Tetra pak - mixed materials	88	0.09%
Aluminium trays	59	0.06%
Metal cans	141	0.14%
Card packaging	1207	1.19%
Glass bottles and jars inc tops	1455	1.43%
Total packaging	4604	4.52%
Unmacerated food - eggshell, pepper top, onionskin	541	0.53%
Total reject stream	5145	5.05%
Food materials - macerated to form SFW	96691	94.95%
Total weight of material	101836	100.00%

### 1.2 Physical appearance, weight and numbers of plastic tokens after digestion

**Fig. 2:**
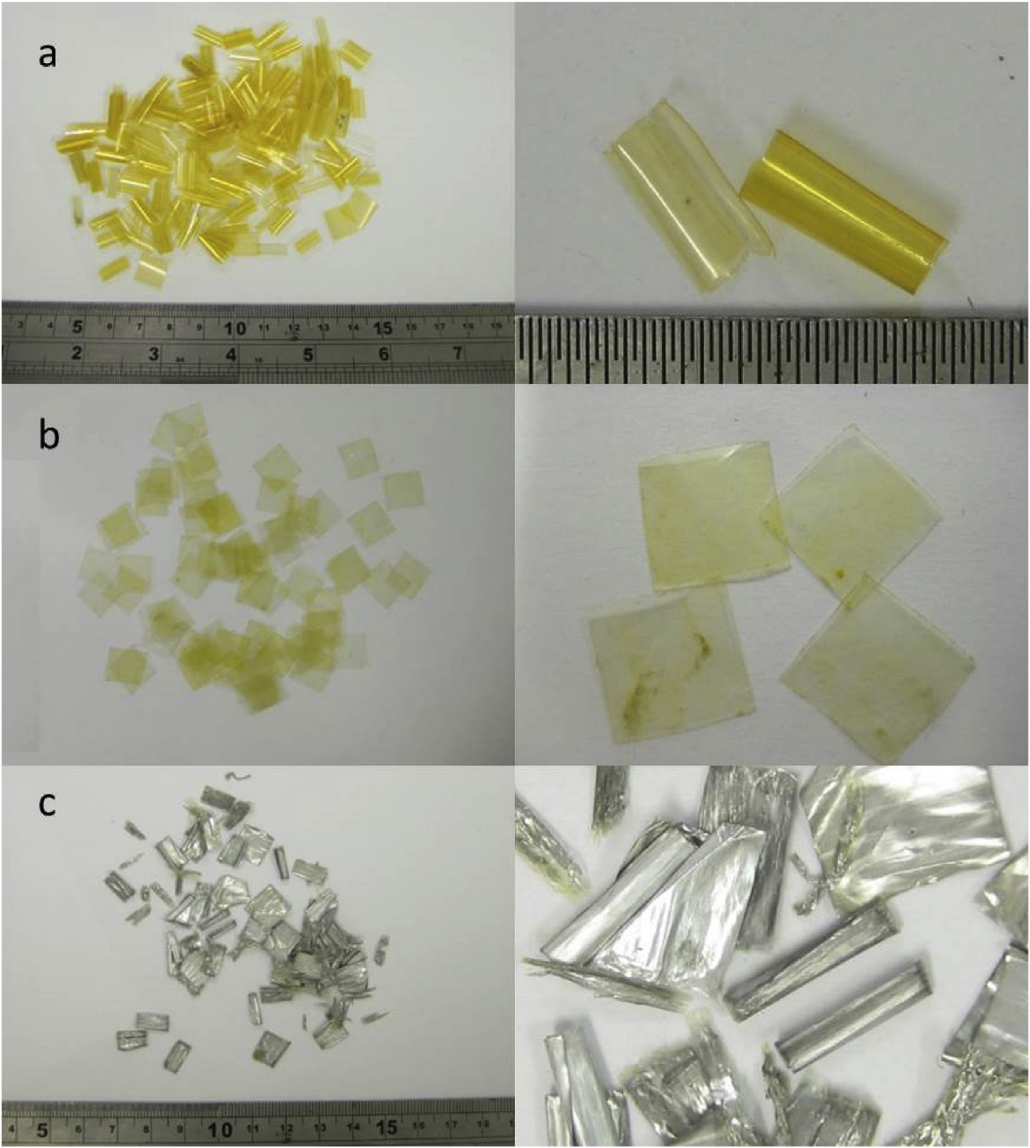

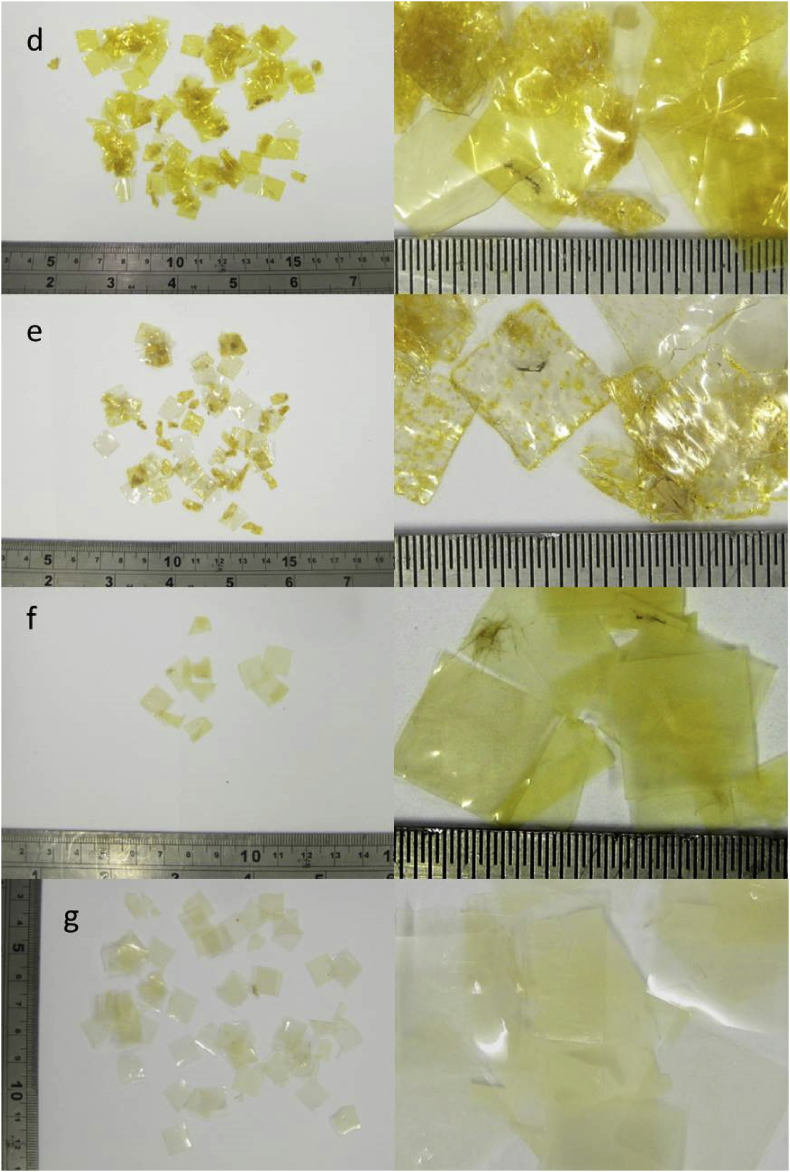

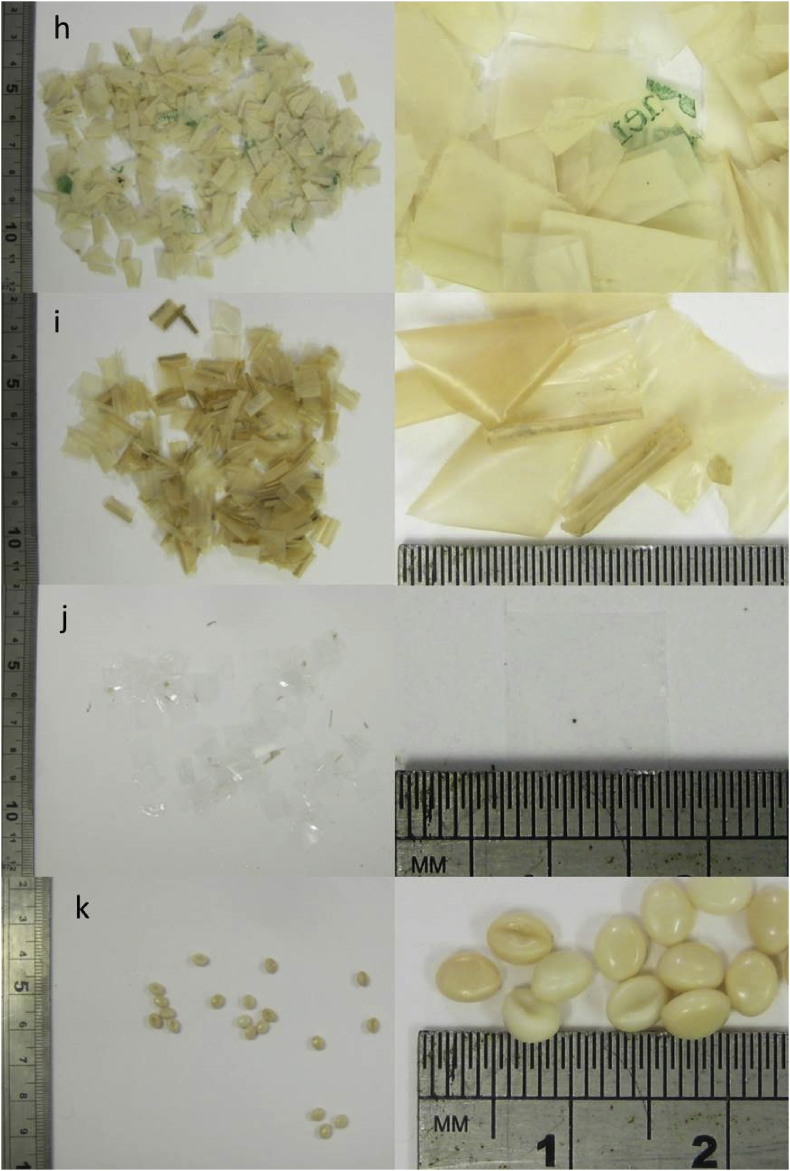
Plastic tokens recovered from digestate samples on day 98 of the digestion trial: (a) PP, (b) LDPE, (c) CBM. (Left-hand image shows total amount recovered in each case). Fig. 2 continued Plastic tokens recovered from digestate samples on day 98 of the digestion trial: (d) CBHS, (e) CBHB, (f) CBnHS, (g) CDF. (Left-hand image shows total amount recovered in each case). Fig. 2 continued Plastic tokens recovered from digestate samples on day 98: (h) SBF1, (i) SBF2, (j) PLAF, (k) PLAB. (Left-hand image shows total amount recovered in each case).

**Fig. 11:**
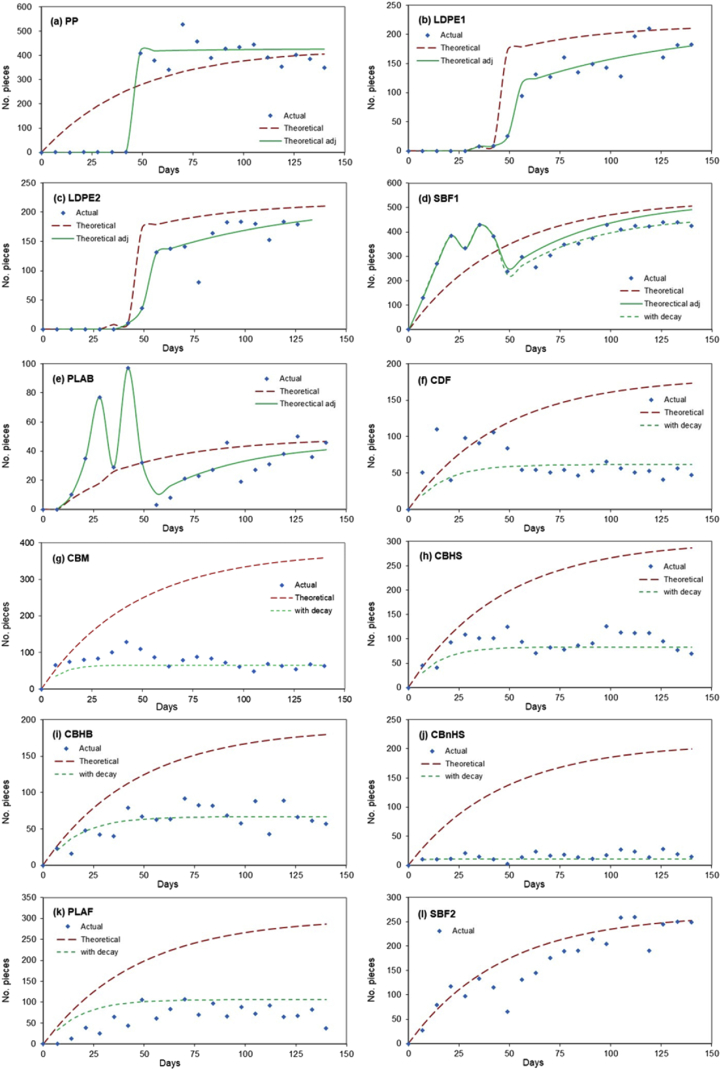
No. of plastic tokens recovered from digestate sample, predicted no. assuming no destruction, and predicted no. modelled using an empirical first-order decay coefficient for (a) PP, (b) LDPE1, (c) LDPE2, (d) SBF1, (e) PLAB, (f) CDF, (g) CBM, (h) CBHS, (i) CBHB, (j) CBnHS, (k) PLAF and (l) SBF2.

Table 2 lists the types of plastic used in the trial in [1]. Fig. 2 shows the plastic tokens removed from the digestate sampled on day 98 of the trial, with the left-hand images showing the total amount recovered in each case. Numbers and weights of tokens during and at the end of the trial are shown in Table 9 and Fig. 11 in Section 2.

**Table 2:** Plastic materials used in trial.

	Abbreviation	Average token weight (mg) 10 × 10 mm square
Polypropylene film	PP	2.61
Low density polyethylene film	LDPE	5.14
Cellulose-based metallised film	CBM	3.42
Cellulose-based heat-sealable film	CBHS	4.28
Cellulose-based high barrier heat-sealable film	CBHB	6.68
Cellulose-based non heat-sealable film	CBnHS	6.24
Cellulose diacetate film	CDF	6.50
Starch-based film blend 1	SBF1	2.17
Starch-based film blend 2	SBF2	4.29
Polylactic Acid Film	PLAF	3.71
		Pellet
Polylactic Acid Blend	PLAB	24.7

**Table 9:** Data for final balance based on no. and weight of tokens and experimentally determined values for degradation constants.

	PP	LDPE	CBM	CBHS	CBHB	CBnHS	CDF	SBF1	SBF2	PLAF	PLAB
No. of tokens added	8906	4293	7884	6278	3942	4380	3796	11096	5548	6278	999
Actual no. of tokens in digester at end	3137	2256	565	918	1038	286	671	3638	1992	1327	320
Actual no. of tokens removed in run	5705	2043	1540	1826	1230	320	1261	7082	3337	1274	655
Predicted total no, of tokens recovered a	3034	1533	466	595	476	76	440	3174	1773	757	297
Actual total no. of tokens recovered	8842	4299	2104	2743	2268	606	1932	10720	5329	2601	975
Balance (no. at end + no. out - no. in)	−64	6	−5780	−3535	−1675	−3774	−1864	−376	−219	−3678	−24
No. of tokens destroyed	0.7%	−0.1%	73.3%	56.3%	42.5%	86.2%	49.1%	3.4%	3.9%	58.6%	2.4%
Weight added (g)	23.29	22.06	26.97	26.89	26.34	27.32	24.68	24.05	23.78	23.31	24.69
Predicted weight in digester at end (g) a	7.93	7.88	1.59	2.55	3.18	0.47	2.86	6.90	7.60	2.81	7.36
Actual weight in digester at end (g)	8.56	10.85	1.67	3.28	5.25	0.98	3.40	7.64	8.69	5.13	7.86
Recovery at end	107.9%	137.7%	104.7%	128.5%	164.9%	206.1%	119.0%	110.8%	114.3%	182.6%	106.8%
Predicted weight removed in run (g) a	15.35	14.19	4.29	6.62	8.04	1.33	7.26	15.24	15.70	7.10	16.58
Actual weight removed in run (g)	15.51	11.38	4.22	5.90	5.97	0.82	6.71	14.50	14.60	4.78	16.08
Recovery in run	101.0%	80.2%	98.3%	89.1%	74.3%	62.0%	92.5%	95.2%	93.0%	67.3%	97.0%
Actual total weight recovered (g) b	24.08	22.23	5.89	9.17	11.22	1.80	10.12	22.14	23.29	9.91	23.93
Actual total weight recovered (%) b	103%	101%	100%	100%	100%	100%	100%	100%	100%	100%	100%
Balance (end + out - in) (g)	0.79	0.16	−21.09	−17.72	−15.12	−25.52	−14.57	−1.91	−0.49	−13.40	−0.76
Weight destroyed	−3.4%	−0.7%	78.2%	65.9%	57.4%	93.4%	59.0%	7.9%	2.1%	57.5%	3.1%
1st order degradation *k*	0.00	0.00	0.10	0.06	0.04	0.39	0.04	0.00	0.00	0.04	0.00
VS destruction potential c	0.0%	0.0%	82.7%	72.3%	64.7%	94.9%	66.2%	12.4%	2.9%	64.8%	6.2%

a Based on 1st-order degradation coefficient.

b Actual total weight recovered = Actual weight in digester at end + Actual weight removed in run.

c Based on value from longer-term modelling with 1st-order degradation coefficient.

### 1.3 Images from microscopy

**Fig. 3:**
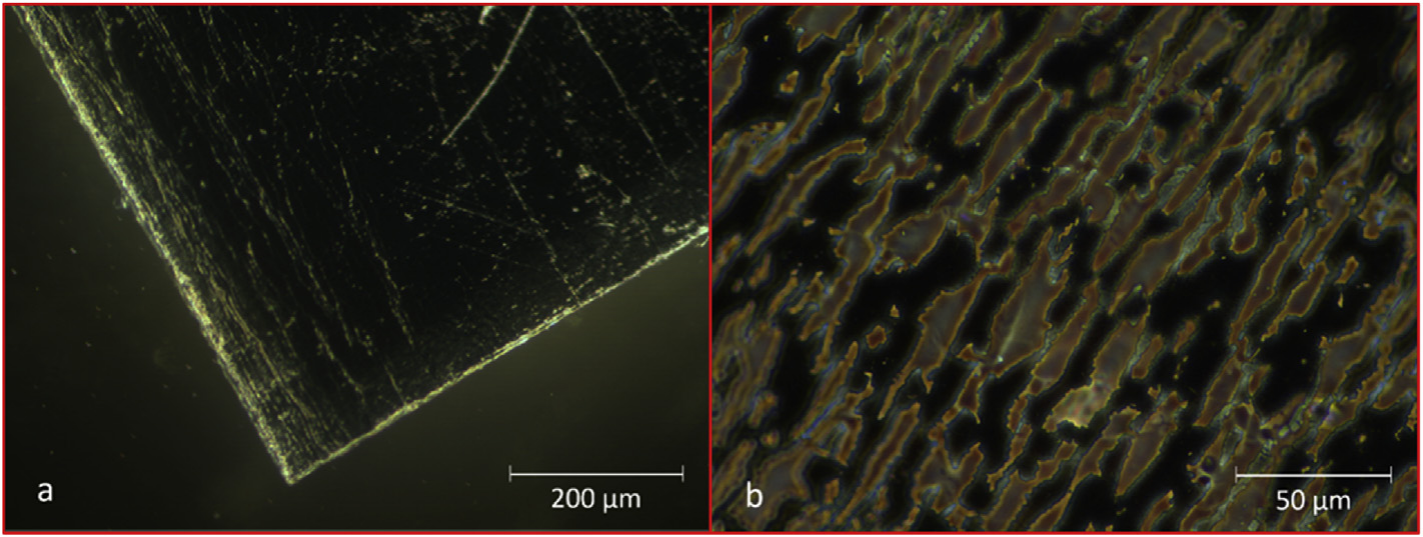
CBM. (a) Low magnification dark field image of CBM film showing areas where the metallic layer has ruptured and is detaching from the surface. (b) Image taken at a higher magnification using phase contrast, showing fractured surface where the metal coating has broken away. Images by Prof Francisco Torrella, University of Murcia.

**Fig. 4:**
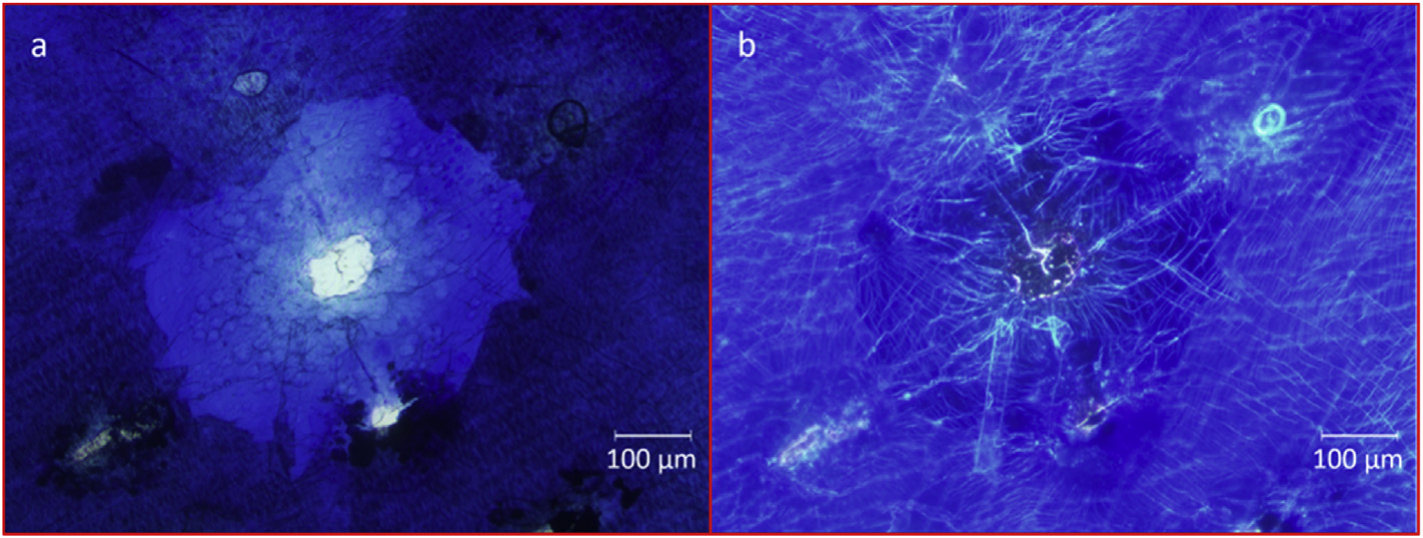
CBM film stained with aqueous methylene blue (MB), showing cellulose beneath the fractured film degrading through the formation of crater-like erosion pits. Bright field image (a) shows darker portions corresponding to areas where metal film is still attached. The reflection of the light in the dark field image (b) of the same area shows details of the material still present at the bottom of the erosion pit, unseen under bright field, with cracks on the film surface as seen from above. Images by Prof Francisco Torrella, University of Murcia.

**Fig. 5:**
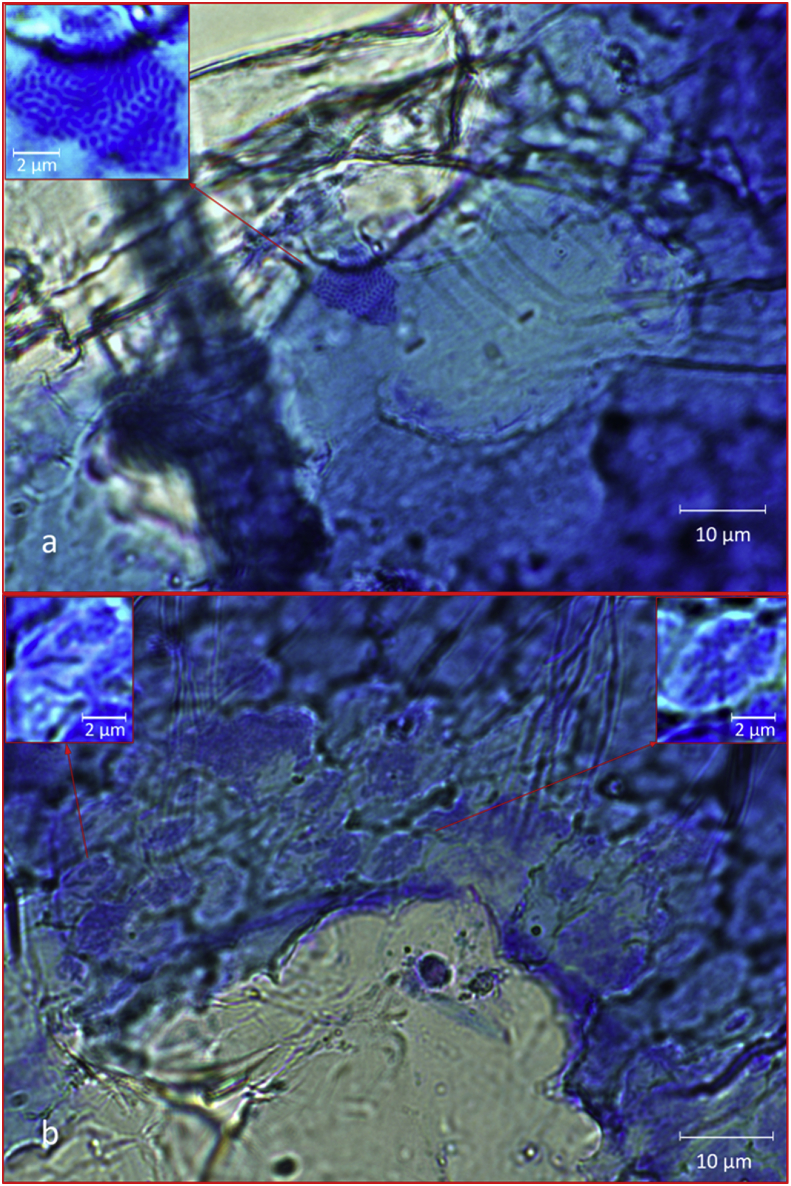
CBM film under bright field (oil immersion 100× objective). (a) Edge of an erosion pit showing bacteria on the pit sides spreading out as a biofilm over a component of the remaining cellulose film. The pinkish-red metachromasy surrounding the clear eroded area in the top left corner is evidence of bacterial growths at the periphery. The depth of focus (approx 0.5 μm) only shows a few bacteria on the borders of the eroded area but visual examination shows bacterial growth extending down into the pit. (b) Image showing bottom of pit and areas of bacterial attack around the edges. Images by Prof Francisco Torrella, University of Murcia.

**Fig. 6:**
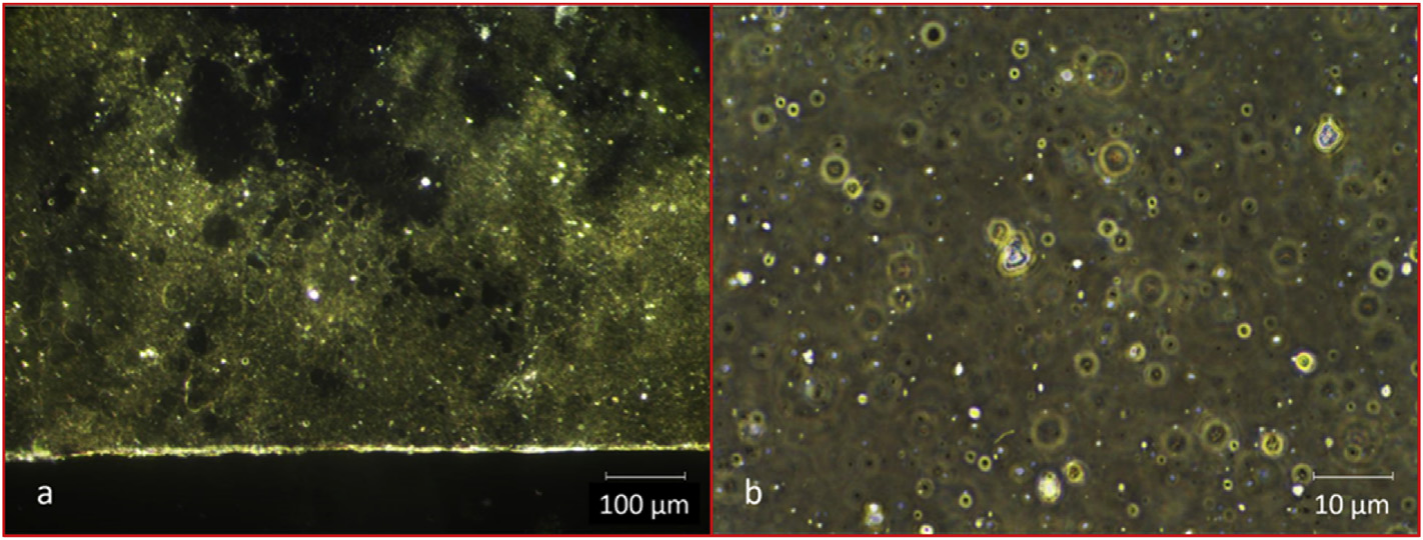
CBnHS. (a) Dark field low magnification clearly showing perforation of film as bright areas where light penetrates thinner sections. (b) Phase contrast showing extensive surface pitting. Images by Prof Francisco Torrella, University of Murcia.

**Fig. 7:**
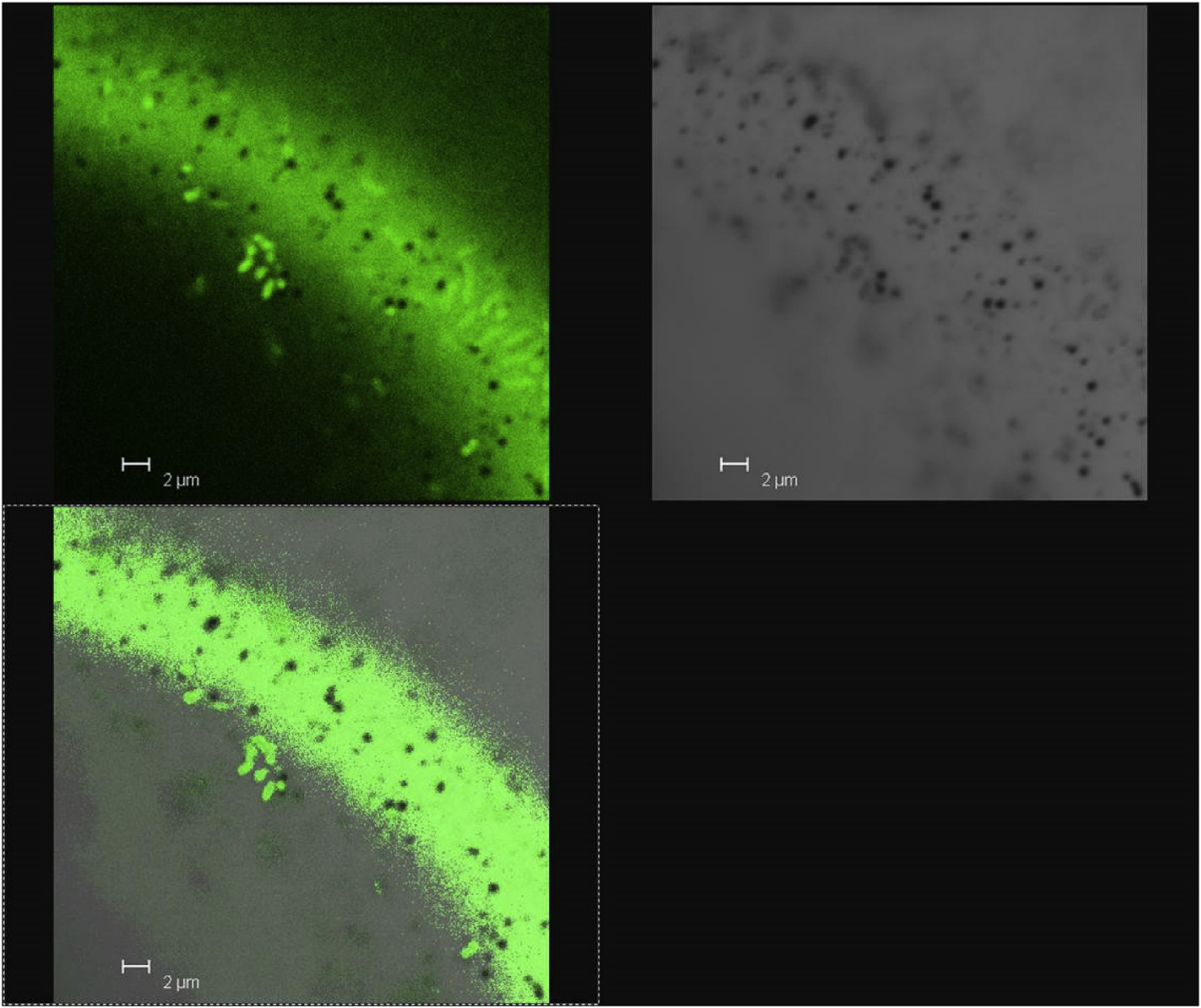
CBHS. Combined fluorescent and differential interference contrast images for sample CBHS showing pitting and microbial attack. Sample viewed using a Leica TCS SP2 confocal laser scanning microscope. Images courtesy of Dr Yue Zhang, University of Southampton.

Fig. 3, Fig. 4, Fig. 5, Fig. 6, Fig. 7 present micrographs of selected plastic pieces recovered from the digestate samples taken on day 98. No special measures were taken to preserve these pieces at the time of sampling. Fig. 3, Fig. 4, Fig. 5, Fig. 6 were taken with light and dark field microscopy and Fig. 7 with confocal microscopy.

### 1.4 Biodegradability of plastics as assessed by the BMP assay

**Fig. 8:**
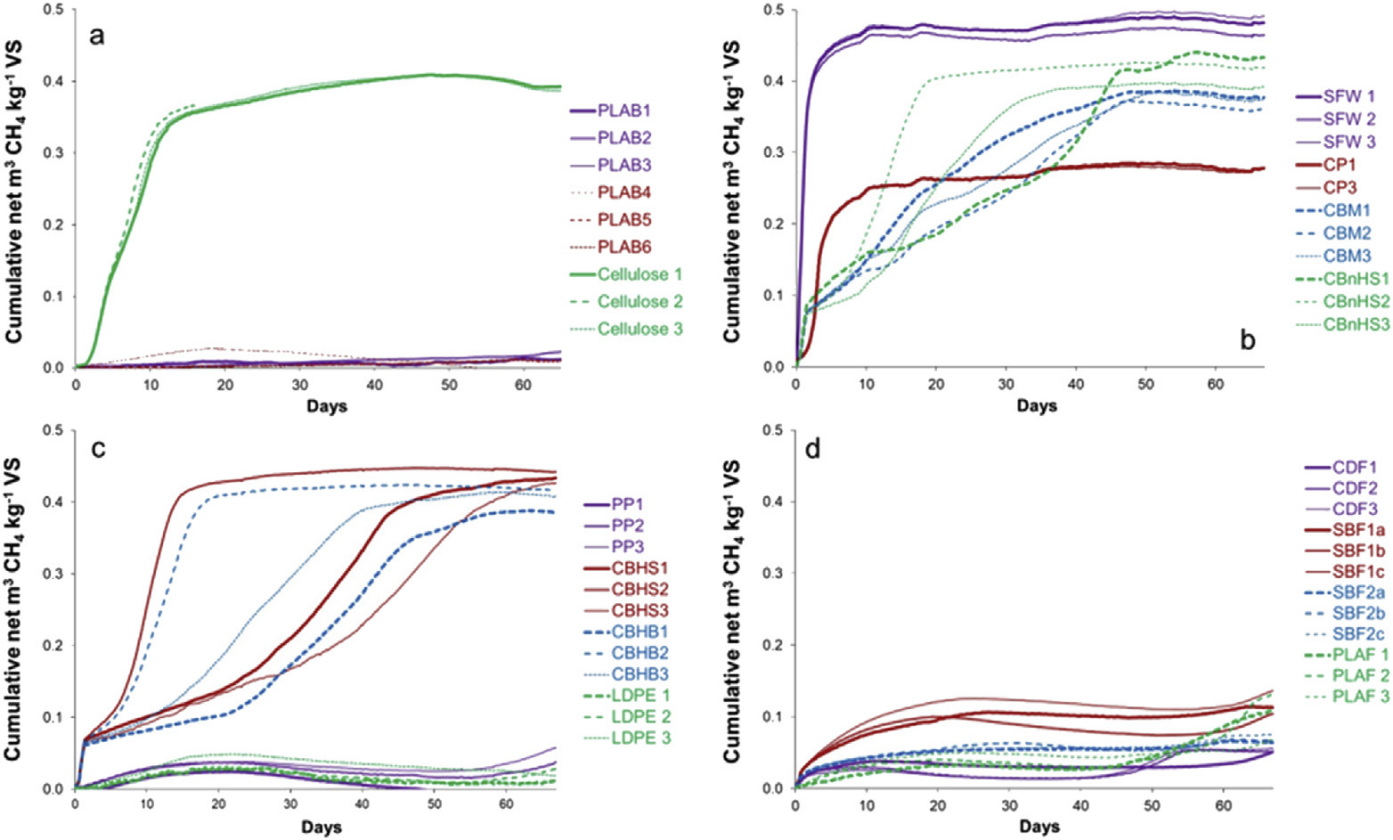
Data from BMP tests on feedstock components: (a) PLAB (1–3 = I/S ratio 3.8, 4–6 = I/S ratio 1.9), cellulose control; (b) SFW, CP, CBM and CBnHS; (c) PP, CBHS, CBHB, LDPE; (d) CDF, SBF1, SBF2, PLAF. I/S ratio = inoculum to substrate ratio used in the assay.

Data from Biochemical Methane Potential (BMP) assays on the feedstock materials (SFW, CP and plastics) used in the trial are shown in Fig. 8 and Table 3. During the BMP assays one replicate for CP and one for PLAB suffered a small loss of digester contents. These replicates were omitted from the BMP calculation and graphical data are presented only up to the point before this loss occurred. Results from another test carried out in accordance with DIN 38414 Teil 8 (high-rate dry fermentation at 50 °C) [2] were made available by the funders of the trial, and are included in Table 3 for comparison.

**Table 3:** 65-day BMP values for plastic samples.

	This work			DIN 8414	DIN 38414	Comments
	m^3^ CH_4_ kg^−1^ VS			m^3^ CH_4_ kg^−1^ VS	days	
PP	0.025	±	0.030	–	–	
LDPE	0.018	±	0.007	0.360	28	
CBM	0.374	±	0.009	0.398	28	DIN 38414 - different grade of CBM
CBHS	0.433	±	0.009	0.340	42	DIN 38414 - not finished
CBHB	0.413	±	0.015	0.397	28	DIN 38414 - almost finished
CBnHS	0.410	±	0.021	0.259	28	
CDF	0.050	±	0.005	0.108	64	
SBF1	0.113	±	0.016	0.069	64	DIN 38414 - not finished
SBF2	0.069	±	0.005	0.058	28	This work - not finished?
PLAF	0.097	±	0.032	0.014	28	
PLAB	0.017	±	0.005	–	–	
Card packaging (CP)	0.274	±	0.046	–	–	
Food waste (SFW)	0.471	±	0.013	–	–	
Cellulose control	0.391	±	0.002	–	–	

Degradation of the cellulose based plastics appeared to show inhibition in the first two days of the BMP assay. Table 4 gives the time of onset of inhibition in each case.

**Table 4:** Onset of inhibition in BMP test for Cellulose-based plastics.

	Onset of inhibition - Days from start of test
CBM	1.49–1.52
CBHS	1.35–1.39
CBHB	1.28–1.30
CBnHS	1.50–1.55

The BMP tests for CDF, SBF1, SBF2, PLAF and PLAB (at both I/S ratios) were left running until day 103. All but PLAF showed little or no change in methane production rate or final yield. PLAF continued to produce methane at a higher rate than in the first 50 days. After 103 days it had produced a further 0.119 m^3^ CH_4_ kg^−1^ VS added, giving a total of 0.216 m^3^ CH_4_ kg^−1^ VS with good agreement between replicates.

### 1.5 Potentially toxic elements

Table 5 shows the concentration of Potentially Toxic Elements (PTE) in the feedstock materials. The method for comparing these with the limit value in the UK's PAS110 standard [3] is outlined in section 2.4.

**Table 5:** Concentration of PTE in feedstock and plastic materials.

	Unit	Mercury (Hg)	Cadmium (Cd)	Chromium (Cr)	Copper (Cu)	Lead (Pb)	Nickel (Ni)	Zinc (Zn)
PAS110 limit value a	kg tonne^−1^ WW	0.08	0.12	8	16	16	4	32
Cardboard	mg kg^−1^ TS	BDL	0.37	4.1	46.8	8.8	2.37	42.8
SFW	mg kg^−1^ TS	BDL	0.02	1.4	3.2	0.08	0.619	17.8
PP	mg kg^−1^ TS	BDL	0.080	0.5	0.4	BDL	0.42	3.0
LDPE	mg kg^−1^ TS	BDL	0.19	0.3	5.4	1.3	0.28	4.1
CBM	mg kg^−1^ TS	BDL	0.693	0.1	0.2	0.2	0.44	4.6
CBHS	mg kg^−1^ TS	BDL	0.06	BDL	BDL	0.2	0.26	1.6
CBHB	mg kg^−1^ TS	BDL	0.04	BDL	BDL	BDL	0.876	0.2
CBnHS	mg kg^−1^ TS	BDL	0.079	0.2	BDL	BDL	0.48	0.4
CDF	mg kg^−1^ TS	BDL	0.12	0.2	0.2	0.1	0.12	1.4
SBF1	mg kg^−1^ TS	BDL	0.064	0.5	2.0	0.2	0.41	1.3
SBF2	mg kg^−1^ TS	BDL	0.16	0.1	0.4	0.1	0.18	2.2
PLAF	mg kg^−1^ TS	BDL	0.15	0.3	1.0	0.4	0.21	1.7
PLAB	mg kg^−1^ TS	BDL	0.068	10.0	BDL	BDL	3.49	0.3

BDL = Below Detection Limit of 0.1 mg kg^−1^ TS.

a PAS110 limit values in kg tonne^−1^ WW at a digestate total N concentration <1 kg N tonne^−1^ WW [3].

### 1.6 Residual biogas potential of digestate

**Fig. 9:**
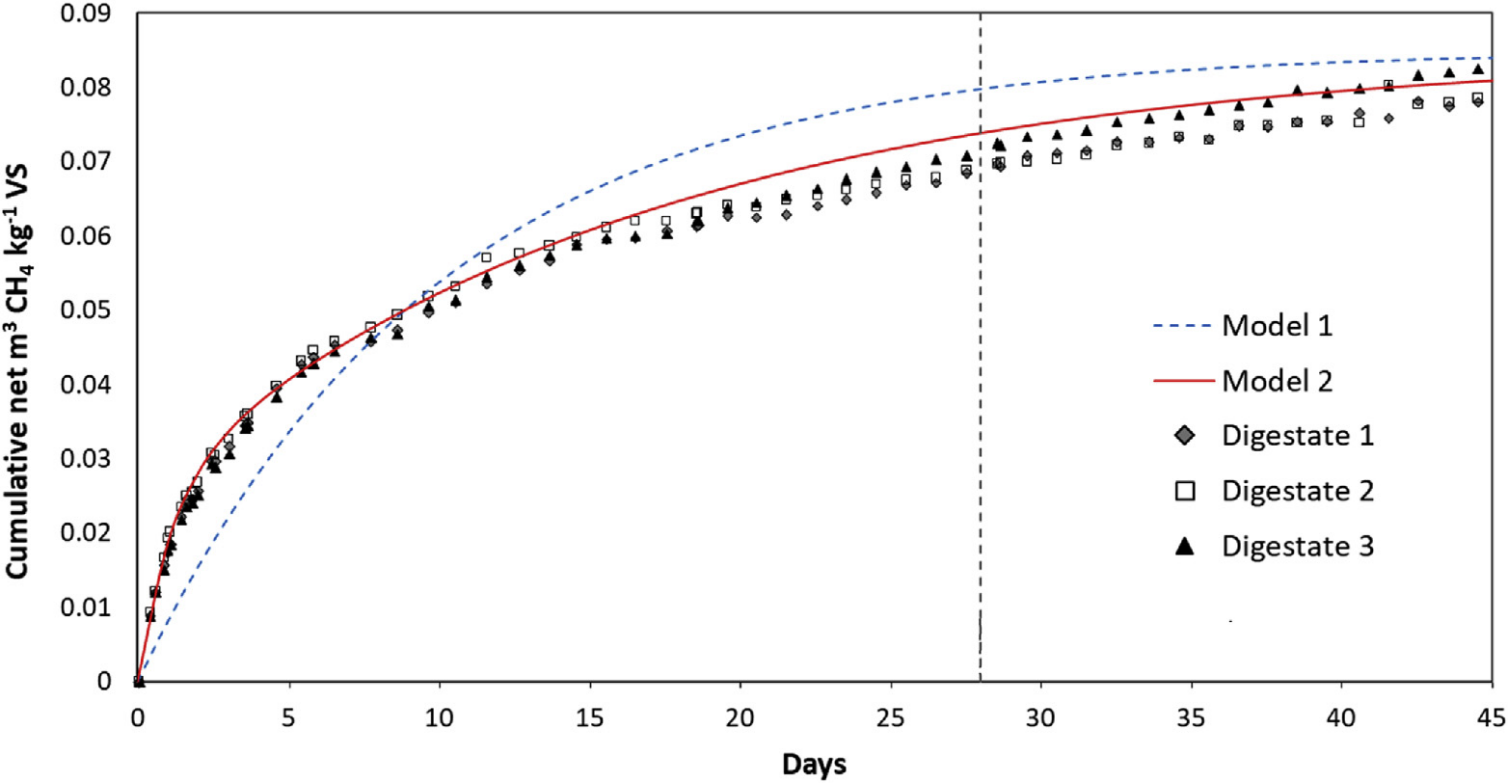
Cumulative net specific methane production from residual whole digestate. Vertical dashed line indicates 28-day test duration.

The Residual Biogas Potential of the digestate from the trial in Ref. [1] was 0.084 L biogas kg^−1^ VS (0.070 L CH_4_ kg^−1^ VS) at day 28. The digestate sample continued to produce gas after the 28-day standard test duration: Fig. 9 shows the data for the cumulative net specific methane production up to day 45. The kinetic constants obtained using two modelling approaches described in Section 2.5 are given in Table 6.

**Table 6:** Kinetic parameters for specific methane yield from digestate.

	Y_m_	P	k_1_	k_2_	R^2^ ave
Model 1	0.085	1	0.10	0.000	0.9796
Model 2	0.085	0.3	0.90	0.060	0.9976

## 2 Experimental design, materials and methods

### 2.1 Synthetic food waste and card packaging

**Fig. 10:**
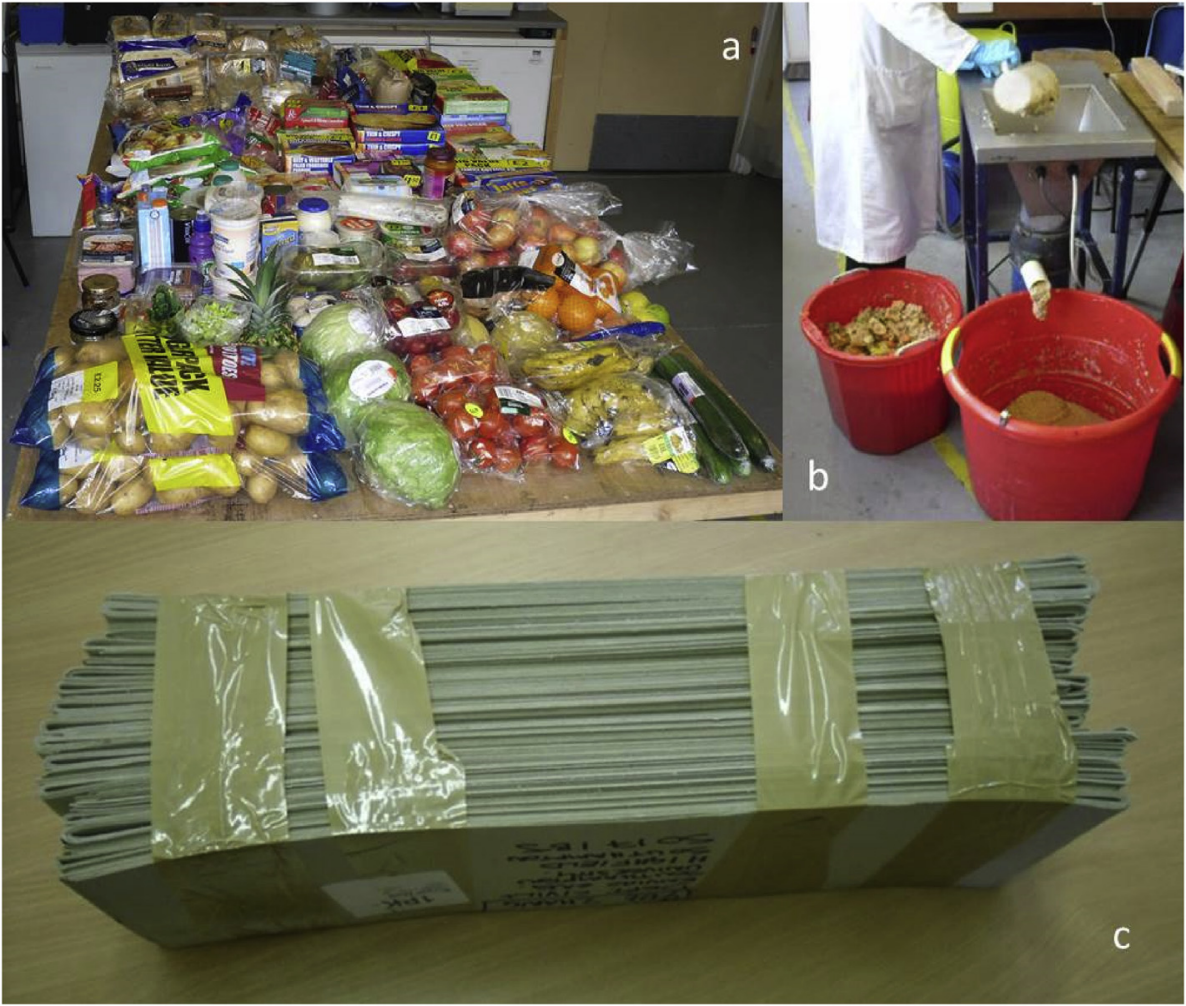
Feedstock materials: (a) Materials purchased for SFW, (b) preparation of SFW by maceration, (c) unprinted card packaging also used in the mixed feed prepared for the trial.

A synthetic food waste, based on materials purchased for the purpose from supermarkets, was prepared for the trial in Ref. [1] as described below. This approach was adopted to ensure that the feedstock for the trial was not contaminated with other plastics, which would have been difficult to avoid using either post-supermarket or post-consumer food waste. A study on post-consumer UK food waste [4] with data categorised into the 100 items most commonly thrown away by households (Table 7) was used as the basis for selection of the materials used. These were further grouped by category according to data provided by a major UK supermarket chain. The selected products were then purchased in appropriate proportions on a fresh weight basis (Table 8), and processed in a macerating grinder (S52/010, IMC Limited, UK) (Fig. 10).

**Table 7:** Most common post-consumer food items for disposal (based on [4]).

No	Item	All (kg)		Short life only (kg)	
1	Potatoes	359000	9.7%	–	0.0%
2	Bread slices	328000	8.9%	328000	11.3%
3	Apples	190000	5.1%	–	0.0%
4	Meat or fish meals	161000	4.4%	161000	5.5%
5	World breads	102000	2.8%	102000	3.5%
6	Veg mixed meals	96000	2.6%	96000	3.3%
7	Pasta mixed meals	87000	2.4%	87000	3.0%
8	Bread rolls/baguettes	86000	2.3%	86000	3.0%
9	Rice mixed meals	85000	2.3%	85000	2.9%
10	Mixed meals	85000	2.3%	85000	2.9%
11	Bananas	84000	2.3%	84000	2.9%
12	Bread loaves	75000	2.0%	75000	2.6%
13	Yoghurts/drinks	67000	1.8%	67000	2.3%
14	Sandwiches	63000	1.7%	63000	2.2%
15	Cakes	62000	1.7%	62000	2.1%
16	Lettuce	61000	1.7%	61000	2.1%
17	Tomatoes	61000	1.7%	61000	2.1%
18	Cabbage	56000	1.5%	56000	1.9%
19	Cooked rice	55000	1.5%	55000	1.9%
20	Mixed veg	53000	1.4%	53000	1.8%
21	Oranges	51000	1.4%	51000	1.8%
22	Carrots	46000	1.2%	46000	1.6%
23	Onions	43000	1.2%	–	0.0%
24	Pears	42000	1.1%	42000	1.4%
25	Sodas	42000	1.1%	–	0.0%
26	Milk	40000	1.1%	40000	1.4%
27	Cheese	40000	1.1%	40000	1.4%
28	Mixed salads	37000	1.0%	37000	1.3%
29	Cooked pasta	36000	1.0%	36000	1.2%
30	Mixed snacks	36000	1.0%	36000	1.2%
31	Melons	35000	0.9%	35000	1.2%
32	Coleslaw	33000	0.9%	33000	1.1%
33	Pizzas	32000	0.9%	32000	1.1%
34	Chicken portions	32000	0.9%	32000	1.1%
35	Cucumbers	32000	0.9%	32000	1.1%
36	Chocolates/sweets	31000	0.8%	31000	1.1%
37	Sweetcorn	30000	0.8%	30000	1.0%
38	Sausages	30000	0.8%	30000	1.0%
39	Pork portions	29000	0.8%	29000	1.0%
40	Biscuits/crackers	27000	0.7%	27000	0.9%
41	Water	27000	0.7%	–	0.0%
42	Beans (not baked)	26000	0.7%	26000	0.9%
43	Grapes	22000	0.6%	22000	0.8%
44	Ham	22000	0.6%	22000	0.8%
45	Plums	20000	0.5%	20000	0.7%
46	Squashes/cordials	20000	0.5%	–	0.0%
47	Breakfast cereals	20000	0.5%	–	0.0%
48	Cook-in sauces	19000	0.5%	–	0.0%
49	Fruit juices	19000	0.5%	19000	0.7%
50	Eggs	19000	0.5%	19000	0.7%
51	Fish	19000	0.5%	19000	0.7%
52	Beef portions	18000	0.5%	18000	0.6%
53	Dough	18000	0.5%	18000	0.6%
54	Celery	17000	0.5%	17000	0.6%
55	Strawberries	16000	0.4%	16000	0.5%
56	Peppers	15000	0.4%	15000	0.5%
57	Chicken drumsticks	15000	0.4%	15000	0.5%
58	Flour	15000	0.4%	15000	0.5%
59	Chicken breasts	15000	0.4%	15000	0.5%
60	Mushrooms	15000	0.4%	15000	0.5%
61	Broccoli	15000	0.4%	15000	0.5%
62	Sandwich spreads	14000	0.4%	14000	0.5%
63	Baked beans	14000	0.4%	–	0.0%
64	Bacon	14000	0.4%	14000	0.5%
65	Peaches	14000	0.4%	14000	0.5%
66	Milk drinks	13000	0.4%	13000	0.4%
67	Crisps	12000	0.3%	12000	0.4%
68	Lemons	12000	0.3%	12000	0.4%
69	Beetroot	12000	0.3%	12000	0.4%
70	Fruit pies	12000	0.3%	12000	0.4%
71	Jams	11000	0.3%	–	0.0%
72	Pheasants	11000	0.3%	11000	0.4%
73	Dips	10000	0.3%	10000	0.3%
74	Mixed fruits	10000	0.3%	10000	0.3%
75	Butter/margarine	10000	0.3%	10000	0.3%
76	Herbs/spices	10000	0.3%	–	0.0%
77	Dessert cakes/gateaux	9000	0.2%	9000	0.3%
78	Cream	9000	0.2%	9000	0.3%
79	Pineapples	9000	0.2%	9000	0.3%
80	Crumpets	9000	0.2%	9000	0.3%
81	Pastry	9000	0.2%	9000	0.3%
82	Chicken products	9000	0.2%	9000	0.3%
83	Pet food	9000	0.2%	–	0.0%
84	Yorkshire pudding and batters	8000	0.2%	8000	0.3%
85	Cauliflowers	8000	0.2%	8000	0.3%
86	Uncooked pasta	8000	0.2%	–	0.0%
87	Leeks	8000	0.2%	8000	0.3%
88	Milk pudding (custards etc)	8000	0.2%	8000	0.3%
89	Doughnuts	8000	0.2%	8000	0.3%
90	Oils	8000	0.2%	8000	0.3%
91	Mayonnaise/salad cream	7000	0.2%	7000	0.2%
92	Spring onions	6000	0.2%	6000	0.2%
93	Peas	6000	0.2%	6000	0.2%
94	Turnips/swedes	6000	0.2%	6000	0.2%
95	Parsnips	6000	0.2%	6000	0.2%
96	Burgers	6000	0.2%	6000	0.2%
97	Lamb	6000	0.2%	6000	0.2%
98	Pickles	6000	0.2%	–	0.0%
99	Nuts	6000	0.2%	6000	0.2%
100	Mangoes	6000	0.2%	6000	0.2%
	Subtotal	3691000	100.0%	2913000	100.0%
	UK total	4080000	90.5%	–	–

**Table 8:** Materials used for preparation of SFW - fresh weight including packaging.

Produce	kg	Bakery	kg	Dry goods	kg	Dairy	kg	Meat and Fish	kg	Ready meals	kg
Potatoes	10.000	White sliced bread	5.650	Bottled water - still	1.700	Yoghurt	2.000	Barbecue mix (sausages, burgers, chicken drumsticks	2.600	Cottage pie	2.000
Apples	6.057	Wholemeal flour	1.740	Potatoes for crisps	1.319	Milk	2.000	Chicken breasts frozen	1.100	Beef lasagne	2.000
Tomatoes	2.518	Sliced wholemeal bread	1.512	Chocolate and confectionery	0.640	Cooked rice	1.175	White fish fillet frozen	0.750	Cooked plain pasta	1.775
Lettuce	2.479	White bread flour	1.500	Mixed breakfast cereal	0.547	Fruit juice	1.000	Breaded chicken breasts	0.640	Pizza	0.930
Bananas	2.270	Pitta bread	1.309	Cook-in sauce	0.540	Coleslaw	0.875	Lamb mince	0.454	Ocean pie	0.900
Oranges	2.048	Wholemeal rolls	1.013	Eggs	0.510	Pasta salad (Chicken/tuna)	0.800	Bacon	0.400	Steak pie	0.800
Mixed vegetables frozen	2.000	Christmas pudding	0.850	Bottled water - sparkling	0.450	Sandwich filling (tuna, onion)	0.750	Ham	0.400	Spinach and ricotta cannelloni	0.600
Melon	1.778	Eggs for cake etc	0.690	Baked beans	0.420	Mayonnaise	0.500	Salami	0.343	Pork pies	0.459
Cucumber	1.525	Tortilla	0.500	Tinned pet food	0.400	Margarine	0.500	Sliced beef	0.100	Spaghetti bolognese	0.450
Pineapple	1.089	Rye bread	0.495	Jaffa cakes	0.300	Custard (liquid)	0.475	–	–	Mushroom Tagliatelle	0.450
Onion	1.009	Apple tart	0.450	Fruit cordial	0.300	Cheddar	0.444	–	–	Stir fry frozen vegetables	0.400
Broccoli mix frozen	1.000	White rolls	0.420	Uncooked pasta	0.250	Fruit dessert	0.400	–	–	Cauliflower cheese grills	0.397
Casserole vegetable mix frozen	1.000	Wholemeal finger rolls	0.400	Granulated white sugar	0.240	Edam	0.320	–	–	Chicken curry	0.375
Sweet corn frozen	1.000	Doughnut	0.330	Jam	0.210	Cottage cheese	0.300	–	–	Beef curry	0.375
Pear	0.860	Crumpet	0.280	Herbs and spices (dry)	0.200	Houmous	0.300	–	–	Chicken curry 2	0.375
Carrots	0.629	Naan bread	0.270	Honey	0.200	Double cream	0.284	–	–	Cheese and onion crisp bakes	0.360
Lemons	0.537	Malt bread rolls	0.230	Mixed nuts	0.200	Brie	0.200	–	–	Beef and yorkshire pudding ready meal	0.360
Celery	0.520	Wholemeal loaf	0.220	Chocolate mini rolls	0.120	–	–	–	–	Vegetable grills	0.340
Grapes	0.500	Water for bread dough	0.200	Chutney	0.100	–	–	–	–	Vegetable lasagne	0.300
Beetroot	0.500	Breadsticks	0.200	Tartare sauce	0.060	–	–	–	–	Yorkshire pudding	0.290
Plums	0.500	Powdered milk	0.100	–	–	–	–	–	–	Stir fry frozen veg	0.400
Pepper	0.498	Gingerbread	0.050	–	–	–	–	–	–	–	–
Peaches	0.433	Yeast	0.015	–	–	–	–	–	–	–	–
Mushrooms	0.350	–	–	–	–	–	–	–	–	–	–
Spring onion	0.160	–	–	–	–	–	–	–	–	–	–
Subtotal	41.260		18.424		8.706		12.323		6.787		14.336
% of total	40.5%		18.1%		8.5%		12.1%		6.7%		14.1%

### 2.2 Semi-continuous digestion trials: adjustment of calculations after amendment of digestate sampling methodology

Semi-continuous digestion trials designed to simulate full-scale operating modes with the addition of plastic tokens were set up and run as described in Ref. [1].

The number and weight of tokens added to each digester, removed each week during the trial, and remaining in each digester at the end of the trial is shown in Table 9. If the sampling method used is representative and the plastic shows little or no degradation, the expected number of tokens removed in any week is simply equal to the number present in the digester multiplied by the fraction of digestate volume removed, and it is easy to keep a running total. For the first weeks of the trial in Ref. [1] the sampling method was not representative, and tended to remove proportionately larger numbers of denser plastic tokens and smaller numbers of less dense tokens. The number of tokens actually removed is still known, however, and if no tokens are lost through degradation the number remaining in the digester at the point when the sampling method was modified can therefore be calculated by simple arithmetic. This value can then be used as the start point for calculating the expected number removed once the sampling method has been adjusted. There are thus two ways to check the assumptions made: firstly, the number of tokens removed or present in the digestate at the end of the run should equal the total number added; and secondly, once the revised sampling method is adopted the number of tokens removed each week should approximately match the expected number.

In the case of the PP control, for example, Table 9 shows that a total of 8906 tokens were added throughout the trial. Of these 8842 were accounted for, either removed with the digestate or present in the digester at the end. Since this material is considered non-degradable, this corresponds to an error of 64 tokens or 0.7% of the total. The equivalent figures for the LDPE control were 4293 tokens with an error of 6 tokens or 0.1%. In Fig. 11 it can also be seen that the expected number of tokens removed showed a reasonably good match to the actual number, once the sampling method had been adjusted and the actual number of tokens present at that point taken into account. This validated the approach used. The same approach could then be applied to plastics such as SBF1 and PLAB, where the number of tokens removed in the first weeks of operation was higher than expected, but the total recovery at the end indicated little or no degradation, as did the other methods of assessment used. In Table 9 it can be seen that the discrepancies in final token numbers for these plastics were 3.4% and 2.4%, only slightly above those for the control plastics; while Fig. 11 again shows good agreement between expected and actual recovery with the adjusted value for tokens once the revised sampling method has been adopted.

This method cannot be reliably applied to more readily degradable plastics without making further assumptions, since the number of tokens recovered is also affected by degradation. The final number and weight of tokens can still be used to estimate the degree of degradation, however. The only readily degradable plastic, which showed clear, signs that a larger than expected number of tokens were being removed during the first few weeks was CDF. In this case no attempt was made to correct the number of tokens present when the sampling method was adjusted (Fig. 11).

### 2.3 BMP test

The conditions used in the BMP assay are described in Ref. [1]. The BMP for a given test substrate was obtained by calculating the cumulative volume of methane produced from each test digester; subtracting the average cumulative STP methane production from the inoculum-only controls; and dividing the result by the weight of substrate volatile solids added to each test digester. The average value in L CH_4_ g^−1^ VS for all test digesters fed on a given substrate was taken as the final BMP value. All gas volumes are reported at STP of 101.325 kPa and 0 °C.

The BMP of the cellulose controls was used to indicate whether the test conditions are satisfactory: the value of 0.391 m^3^ CH_4_ kg^−1^ VS added in this case was very close to the theoretical value of 0.3415 m^3^ CH_4_ kg^−1^ VS added. The SFW and CP had BMP values of 0.471 and 0.274 m^3^ CH_4_ kg^−1^ VS added respectively, both typical of these types of material. The control plastics PP and LDPE showed very low but non-zero values of 0.025 and 0.018 m^3^ CH_4_ kg^−1^ VS added respectively, corresponding to around 5% of the methane yield of the controls and indicating the probable limit of accuracy of the assay.

The data for the cellulose-based plastics were not ideal for the purposes of determining the BMP and the calculation was thus adapted to accommodate this. All four plastics produced methane at a rapid and consistent rate from the start of the test until between 1.2 and 1.5 days (Fig. 8b and c), when methane production relative to the inoculum-only controls dropped sharply. Inhibition of this type is often due to production of volatile fatty acid (VFA) intermediates at a rate greater than the capacity of the methanogenic population to process the VFA into methane, and this in turn indicates a very readily degradable material and an insufficient I/S ratio in the test. To confirm the cause would require sampling an additional replicate to measure system parameters such as pH, alkalinity and VFA concentration, but this was not carried out in the current work. An alternative explanation of some inhibitory component in the heat-sealable and moisture-resistant surface layers of the plastics was ruled out, as the same effect also occurred in CBnHS without these additional layers. The onset of inhibition appeared to be a characteristic of the material, as there was little overlap between the different plastics (Table 4). Unfortunately recovery from this type of inhibition generally shows considerable variation between replicates, and can have some impact on the final BMP value, as seen in Fig. 8b and c. The outlying values for CBM, CBHB and CBnHS were therefore ignored in calculating the average BMP for each material. Despite this issue, the BMP values showed reasonable correspondence with those obtained from the DIN 38414 test (Table 3), especially when the degree of completion of some of the DIN 38414 test runs is taken into account.

Of the remaining plastics, SBF2 showed a very low BMP of 0.069 m^3^ CH_4_ kg^−1^ VS added, while SBF1 had a slightly higher value of 0.113 m^3^ CH_4_ kg^−1^ VS added. In both cases the similarity to DIN 38414 test values may be coincidental, as gas production was still continuing at a low but steady rate at the end of the DIN 38414 test. For CDF film there was a considerable difference between the value of 0.05 m^3^ CH_4_ kg^−1^ VS added in this work and the DIN 38414 test value of 0.259 m^3^ CH_4_ kg^−1^ VS added, suggesting that this material may be more amenable to degradation under thermophilic conditions than in a wet mesophilic system. The BMP value in this work of 0.097 m^3^ CH_4_ kg^−1^ VS added for PLAF was higher than the DIN 38414 test value, but the DIN 38414 test ran for only 28 days and gas production was continuing steadily at the end (Table 3). In the current work there appeared to be a slight increase in methane production from PLAF from day 50 onwards (Fig. 8d). On the basis of this, the BMP tests for CDF, SBF1, SBF2, PLAFand PLAB (at both I/S ratios) were left running until day 103.

### 2.4 Potentially toxic elements

Potentially Toxic Elements in the plastic samples were measured by NRM Ltd. The limiting factor for plastic addition can be determined by comparison with the permissible loadings under the UK's PAS110 standard [3], in which application rates are based on the total nitrogen content of the digestate. The following simple assumptions were made to assess this. If a digester were fed on 100% plastic and achieved a 95% degradation rate, then only one material (PLAB) would exceed the standard for chromium and nickel, with five others (CBM, CBnHS, CDF, SBF2 and PLAF) slightly exceeding the cadmium standard. In practice however the concentration of plastic in a mixed feedstock is unlikely to exceed 2%, and degradation rates are generally below 95%. At the bioplastics loading required for compliance with the PAS110 physical contaminants specification, for example, the materials could not cause the digestate to exceed the specified limit values for PTE. The determining factor for metals concentrations in the digestate will therefore be that in the food waste and card packaging components.

### 2.5 Methodology for residual biogas potential of digestate

In order to determine whether the mixed whole digestate from the trial in Ref. [1] was likely to meet the requirements of the PAS110 standard [3], one of the duplicate LDPE control reactors was sacrificed on day 126 and the digestate was tested for residual biogas production (RBP). The test was carried out in triplicate in static reactors with a sewage sludge inoculum according to the methodology used in OFW004-005 (2009) [5]. To provide additional information on the stability of the material, the methane content of the biogas was also measured to give a static batch test BMP value.

To determine kinetic constants, the specific methane production was modelled using two sets of assumptions: simple first-order degradation (Model 1), and a pseudo-parallel first-order model (Model 2). For model 1 the methane production is given by

(1) Y = Y_m_ (1 - e^-kt^)

Where.

Y is the cumulative methane yield at time t.

Ym is the ultimate methane yield.

k is the first order rate constant.

Rao (2002) [6] suggests that for certain materials it may be better to consider that the gas production curve corresponds to the rapid breakdown of readily degradable components followed by a much slower degradation of the remaining material. The methane production is therefore governed by two rate constants k_1_ and k_2_ rather than by a single constant:

(2) Y = Y_m_ (1 - Pe^-k^_1_^t^ - (1-P) e^-k^_2_^t^)

Where:

Y is the cumulative methane yield at time t.

Y_m_ is the ultimate methane yield.

k_1_ is the first order rate constant for the proportion of readily degradable material.

k_2_ is the first order rate constant for the proportion of less readily degradable material.

P is the proportion of readily degradable material.

Model 1 gave only a moderately good fit to the data (R^2^ ≈ 0.98). A much better fit was obtained using model 2 (R^2^ ≈ 0.998), especially in the early stages of the digestion period. The data showed that while the material is depleted it still contains a more rapidly-degradable fraction, as expected for a fully-mixed system.

The estimated final BMP value of 0.085 m^3^ CH_4_ kg^−1^ VS added was compared with limit value of 0.45 L biogas kg^−1^ VS in the UK's PAS110 [3] to confirm that digestate would meet the standard and be suitable for disposal. The 45-day residual methane production of 0.087 m^3^ CH_4_ kg^−1^ VS from the CSTR trial was compared with the static BMP test and showed good agreement. The 45-day biogas yield of 0.137 m^3^ kg^−1^ VS reflects the absence of losses due to CO_2_ dissolution using this method, compared to methods involving collection under a barrier solution.

## Funding sources

The work was commissioned and funded by the UK's National Non-Food Crops Centre.
